# Development and application of a method to detect 27 respiratory pathogens using multiplex RT-PCR combined with MassARRAY technology

**DOI:** 10.1186/s12879-021-06404-0

**Published:** 2021-08-25

**Authors:** Huan Zhao, Yichao Yang, Jiangfeng Lyu, Xuyi Ren, Wei Cheng

**Affiliations:** 1 Department of Respiratory Medicine, The Sixth People’s Hospital of Nantong, Nantong, Jiangsu China; 2 Research and Development Centre, Hangzhou DiAn Medical Laboratory, Hangzhou, Zhejiang China; 3 Department of Respiratory Diseases, Nantong Tongzhou People’s Hospital, Nantong, Jiangsu China

**Keywords:** Respiratory pathogens, Multiplex RT-PCR, MassARRAY technology

## Abstract

**Background:**

Respiratory tract infections are the most common infections that lead to morbidity and mortality worldwide. Early recognition and precise diagnosis of microbial etiology is important to treat LRTIs promptly, specifically and effectively.

**Objectives:**

To establish a method based on multiplex reverse transcription (MRT)-PCR and MassARRAY technology for the simultaneous detection of 27 respiratory pathogens and explore its clinical application value.

**Methods:**

Analytical sensitivity and specificity of the MRT-PCR-MassARRAY system were validated using inactivated bacterial and viral strains. Also we analyzed samples from 207 patients by MassARRAY methods and compared the results with consensus PCR/reverse transcription (RT)-PCR.

**Results:**

The minimum detection limit of our MRT-PCR-MassARRAY method for pathogens was 10–100 copies/μl, with high specificity. Comparison test with consensus PCR/RT-PCR on 207 clinical samples, the positive, negative, and total correlation rates were 100, 98.68, and 99.03%, respectively. There was a high degree of agreement between the test results of the two methods (*P* < 0.01 by McNemar’s test).

**Conclusion:**

Our detection system of 27 respiratory pathogens based on MassARRAY technology has high sensitivity and specificity, high throughput, and is simple to operate. It provides diagnostic value for the clinical diagnosis of respiratory pathogens and is of great significance in the screening of respiratory pathogens.

## Background

Respiratory tract infections are common infectious diseases in clinics. They are highly prevalent in infants, the elderly and immunocompromised patients, often leading to severe symptoms or mortality [1]. According to the WHO, approximately 3 million people died from lower respiratory tract infections in 2016 [2]. Common pathogens of respiratory tract infections include viruses, bacteria, mycoplasmas, chlamydiae, and other microorganisms [3]. Viral pathogens mainly include influenza A virus, influenza B virus, parainfluenza virus (PIV), respiratory syncytial virus, rhinovirus, human metapneumovirus (HMPV), and adenovirus [4]. Bacterial pathogens include *Streptococcus pneumoniae*, *Haemophilus influenzae*, and *Moraxella catarrhalis* [5]. Most respiratory tract infections have similar clinical symptoms, making their identification difficult for clinicians. Thus, the rapid identification of pathogens of respiratory tract infections is of great significance for accurate clinical diagnosis and the administration of appropriate medication. Over the past few years, the rapid development of molecular detection technology has largely compensated for the shortcomings of traditional culture and immunoassay methodologies used to detect respiratory pathogens, substantially improving the speed, sensitivity, and specificity of the detection and identification of respiratory pathogens. In particular, the development of pathogen detection systems based on multiplex PCR allows the simultaneous detection of many different pathogens for use in clinical applications [6–9]. Multiplex real-time quantitative fluorescence PCR detection has relatively high sensitivity and specificity but is limited by the number of fluorescence types in each reaction. Generally, only 3–4 pathogens can be detected in one reaction, and if more pathogens need to be detected, more reactions are required, substantially reducing the convenience of detection and throughput. Luminex’s NxTAG Respiratory Viral Panel is based on liquid chip technology and can simultaneously detect 20 pathogens, including 18 viruses and subtypes, as well as *Mycoplasma pneumoniae* and *Chlamydia pneumoniae* [10]. However, this commercial kit has not been widely used in clinics due to the high cost of its reagents.

Agena Bioscience’s MassARRAY System integrates the sensitivity of PCR technology and the high throughput of mass spectrometry detection technology. One reaction can achieve up to 40 gene amplifications that can be applied to single nucleotide polymorphism analysis, mutant detection, DNA methylation analysis, gene copy number identification, and other research areas [11]. Currently, the main applications of MassARRAY in clinics are deafness gene detection, epidermal growth factor receptor mutation detection, liquid biopsy, and hypertension gene detection [12–15]. In this study, we developed a new method based on multiplex reverse transcriptase (MRT)-PCR combined with MassARRAY technology to detect 27 common pathogens causing respiratory tract infections. We also evaluated its application value in clinics in the diagnosis of pathogens of respiratory tract infections.

## Methods

### Sample collection

We collected nasopharyngeal aspirate samples from 207 patients with suspected respiratory tract infection who were submitted to the sixth people’s hospital of Nantong from September 2019 to March 2020. The patients were aged 1–76 years only and comprised 112 males and 95 females. The collection of all NPA samples was approved by the ethics committee of the sixth people’s hospital of Nantong. And informed consent was obtained from all subjects. We confirmed that all methods in this study were carried out in accordance with relevant guidelines and regulations.

### Primers used for multiplex PCR and extension reaction

Specific amplification primers and extension primers for all targets including several influenza A virus (Flu-A H1N1, H3, H5, H7, H3N2 seasonal type), influenza B virus (Flu-B), parainfluenza virus types 1 to 4 (PIV1 to 4), four human coronaviruses (HCoV-OC43, 229E, NL63, and HKU1), respiratory syncytial viruses (RSV), human metapneumoviruses (HMPV), human bocavirus (HBoV), human rhinovirus (HRV), human enterovirus (HEV), adenovirus (ADV), *Moraxella catarrhalis* (*M. catarrhalis*), *Mycoplasma pneumoniae* (MP), *Chlamydia pneumonia* (CP), *Bordetella pertussis* (*B.pertussis*), *Haemophilus influenza*(*H.influenza*), *Streptococcus pneumoniae(S. pneumoniae), Streptococcus pyogenes (S.pyogenes), Streptococcus agalactiae (S.agalactiae)* and *Legionella pneumophila (L.pneumophila*) were designed by MassARRAY Assay Design software using published sequences from Gene Bank (Table 1). All primers were synthesized by Invitrogen Corp (Shanghai, China).

**Table 1:** Sequences of amplification primers for 27 pathogens

Pathogen	Forward Primer (5 → 3)	Reverse primer (5 → 3)
*M.catarrhalis*	ACGTTGGATGTAGCAGGCGGTGTTGGTGTT	ACGTTGGATGTTGTAGCTTGCTTCAACGCC
HRV	ACGTTGGATGAACAGTGGTCCAGCCTGCGT	ACGTTGGATGAACACGGACACCCAAAGTAG
MP	ACGTTGGATGCCCGAACAAAATAATGATTCC	ACGTTGGATGGTTTGACAAAGTCCGTGAAG
HCoV	ACGTTGGATGGGGAGTAATGAACCCGGTAA	ACGTTGGATGGAGCTAATAACACGGCTCTG
Flu-A	ACGTTGGATGAAGACCAATCCTGTCACCTC	ACGTTGGATGAAAGCGTCTACGCTGCAGTC
CP	ACGTTGGATGCGGAAGGGTTAGTAGTACAT	ACGTTGGATGCGCATAAACTCTTCCTCAAC
Flu-B	ACGTTGGATGACGGTGGATTAAACAAAAGC	ACGTTGGATGGCAATAGCTCCGAAGAAACC
HMPV	ACGTTGGATGAGCTTCAGTCAGTTCAACAG	ACGTTGGATGCCTGCAGATGTTGGCATGTA
*B. pertussis*	ACGTTGGATGTGGGAGTTCTGGTAGGTGTG	ACGTTGGATGATCAAGCACCGCTTTACCC
*H. influenzae*	ACGTTGGATGTTGGCCCAGGTTGGTATATG	ACGTTGGATGTTACGCACGGTAAGGATG
H5	ACGTTGGATGTGACTATCCACAATACTCAG	ACGTTGGATGGACCAGCTATCATGATTGCC
*S.pyogenes*	ACGTTGGATGAGCAACAAGTAGTACAGCAG	ACGTTGGATGTTAGCTACTAGTGTAGCTG
*S.pneumoniae*	ACGTTGGATGTCGTGCGTTTTAATTCCAGC	ACGTTGGATGACGCAATCTAGCAGATGAAG
PIV4	ACGTTGGATGGATCCACAGCAAAGATTCAC	ACGTTGGATGCCTGCAAGGAAAGCAGAGAT
H1N1	ACGTTGGATGGTTAACATCAGCAACACCAAC	ACGTTGGATGAGAGAGGAATTGCCCGC
*S.agalactiae*	ACGTTGGATGGAAACATTGATTGCCCAGC	ACGTTGGATGAGGAAGATTTATCGCACCTG
PIV3	ACGTTGGATGACTCTATCCACTCTCAGACC	ACGTTGGATGATGGGATCTGAGGATAC
PIV2	ACGTTGGATGCTGCAGCTATGAGTAATCAC	ACGTTGGATGGATCGAGCATCTGGAATAAC
ADV	ACGTTGGATGGACGCCTCGGAGTACCTGA	ACGTTGGATGAGCCACCGTGGGGTTTCTAA
RSV	ACGTTGGATGCACAGAAGATGCTAATCAT	ACGTTGGATGGGTCTTCTCTTCCTAATC
H3	ACGTTGGATGCAGTACAGGGAATCTAATTG	ACGTTGGATGGCATTCAGAATTGCATTTG
*L.pneumophila*	ACGTTGGATGTGGTGACTGCAGCTGTTATG	ACGTTGGATGTCCGGATTAACATCTATGCC
PIV1	ACGTTGGATGATTACCTGGACCAAGTCTAC	ACGTTGGATGCACATCCTTGAGTGATTAAG
HEV	ACGTTGGATGCTGAATGCGGCTAATCCCAA	ACGTTGGATGGATTGTCACCATAAGCAGCC
H7	ACGTTGGATGCAGCATACAATTGATCTGGC	ACGTTGGATGCCATCTTCTTCAGCATTCTC
H3N2 seasonal	ACGTTGGATGACCACCCGGGTACGGACAA	ACGTTGGATGGGCTTCTTTTGGTAGATACTG
HBoV	ACGTTGGATGAAATCTCTTCTGGCTACACG	ACGTTGGATGCCTCTGCGATCTCTATATTG
GAPDH	ACGTTGGATG CTGCTCACATATTCTGGAGGAG	ACGTTGGATG AAAAGCAGCCCTGGTGACC

### Nucleic acids extraction

Extraction of nucleic acids (NA) from 200 μl of NPA was performed by MagNA Pure LC extraction using the total nucleic acid extraction kit (Roche Diagnostics, Penzberg, Germany), and the operation steps were performed in strict accordance with the instructions of the kit. Sample quality control was evaluated by detection of the glyceraldehyde 3-phosphate dehydrogenase (GAPDH) gene as an internal control.

### Multiplex RT-PCR and MassARRAY detection

First, we performed MRT-PCR using a 40 μl reaction mix contained 4 μl 10 × PCR buffer (Takara, Japan), 0.25 mM dNTP (Takara), 4 mM MgCl2, 0.5 μM of each amplification primer, 20 u PrimeScript™ II reverse transcriptase (Takara), 1u TaKaRa Hot start-Taq polymerase. The MRT-PCR was carried out on AB 2720 PCR instrument (AB, USA) for thermocycling and the cycling conditions were 50 °C 25 min, 95 °C 5 min, followed by 45 cycles of (94 °C 15 s, 55 °C 30 s, 68 °C 20 s). Second, we used shrimp alkaline phosphatase to remove dNTPs from the PCR product (dephosphorylation), 5 μl RT-PCR product from the first step was taken off and mixed with 2 μl shrimp alkaline phosphatase (Agena, USA), incubated at 45 °C for 25 min, 80 °C for 2 min. Third, we added 2 μl of single-base extension solution (Agena Bioscience) and 0.5 μM of each extention primer to the product of the second step, and then performed single-base extension as follows: 94 °C for 30 s, followed by 30 cycles of (94 °C for 5 s, five cycles of [52 °C for 5 s, 80 °C for 5 s]), and 72 °C for 3 min. After resin desalination, 10 μl of the extended PCR product was loaded onto the MassARRAY chip (Agena Bioscience), and the mass spectrometry results were analyzed with Typer 4.0 software (Agena Bioscience).

### Sensitivity and specificity assessment of MRT-PCR-MassARRAY

The sensitivity of the MRT-PCR-MassARRAY for pathogen detection was studied using inactivated bacterial and viral strains with a quantitative certification, which were purchased from National Institutes for Food and Drug Control, China. The strains underwent serial dilution to obtain 10,000, 1000, 100, and 10 copies/μl. The detection results were automatically interpreted by the instrument. A positive result was indicated by a reduced or totally consumed extension primer peak and the extension product peak amplifying one base at the corresponding position. The extension primer peak sequence, molecular mass of each pathogen, and the extension product sequence and molecular mass are shown in Table 2. Each diluted pathogen was tested in triplicate, and the lowest dilution concentration that was positive in all three replicates was defined as the limit of detection. We investigated the specificity of the detection system by performing MRT-PCR on 10,000 copies/μl of each pathogen. If each pathogen extension product peak occurred at a specific location and there was no cross-overlap between each pathogen extension product peak, the specificity of the detection system was considered to be good.

**Table 2:** Sequence and molecular mass of extension primer and extension product

Pathogen species	Extension Primer Sequence	Molecular mass of extension primer	Extended base	Extension Product Sequence	Elongation Product Molecular Mass
*M.catarrhalis*	TCAACGCCCACATTT	4471.9	G	TCAACGCCCACATTTG	4759.1
HRV	CTTTGAGTCCTCCGGC	4824.1	C	CTTTGAGTCCTCCGGCC	5071.3
MP	AAAGCCACCCTGATCAC	5108.4	C	AAAGCCACCCTGATCACC	5355.5
HCoV	ACGGCTGTGTAAAGA	5234.4	T	ACGGCTGTGTAAAGAT	5561.5
Flu-A	CACGCTGCAGTCCTCGCT	5411.5	C	CACGCTGCAGTCCTCGCTC	5658.7
CP	TTCCTCAACCGAAAGGTC	5443.6	C	TTCCTCAACCGAAAGGTCC	5690.7
Flu-B	AAAGGCCATAGGGAATTG	5596.7	C	AAAGGCCATAGGGAATTGC	5843.9
HMPV	ACGCGGCAGTTTTCAGACA	5812.8	A	ACGCGGCAGTTTTCAGACAA	6084
*B. pertussis*	CTTCCCGCCCAGACCAAT	5966.9	G	CTTCCCGCCCAGACCAATG	6254.1
*H. influenzae*	GATGCACTTCCACATTATAT	6051	T	GATGCACTTCCACATTATT	6378.1
H5	GATAAACAGTGGCGAGTTCC	6166	C	GATAAACAGTGGCGAGTTCCC	6413.2
*S.pyogenes*	CCCCCTCCAGGAGCAACTTGA	6336.1	G	CCCCCTCCAGGAGCAACTTGAG	6623.3
*S.pneumoniae*	GTATCAGATGAAGCAGGTTTG	6525.3	C	GTATCAGATGAAGCAGGTTTGC	6772.4
PIV4	GGTAGTAATGCCCCTTGCTTAA	6725.4	T	GGTAGTAATGCCCCTTGCTTAAT	7052.5
H1N1	CTGTACAGTCAGTGGTTTCCGT	6732.4	G	CTGTACAGTCAGTGGTTTCCGTG	7019.6
*S.agalactiae*	CCAGGCCCCCCACGATACTCATG	6914.5	C	CCAGGCCCCCCACGATACTCATGC	7161.7
PIV3	GATCTCTGAGGATACAGATGAAT	7111.7	G	GATCTCTGAGGATACAGATGAATG	7398.9
PIV2	CCCTGTTATTTCTACTCTATCTAT	7194.7	G	CCCTGTTATTTCTACTCTATCTATG	7481.9
ADV	CCCCCAAGTACGTGTCGGTGGCAC	7314.7	G	CCCCCAAGTACGTGTCGGTGGCACG	7602
RSV	CTATTCTCTTCCTAATCTAGACATA	7525.9	G	CTATTCTCTTCCTAATCTAGACATAG	7813.1
H3	CGCTAAATAATGAGATCAGATGCAC	7683	C	CGCTAAATAATGAGATCAGATGCACC	7930.2
*L.pneumophila*	GTATTTTTAAAATTCTTCCCCAAATC	7854.1	C	GTATTTTTAAAATTCTTCCCCAAATCG	8141.3
PIV1	GGGACGAATACGCATATTGCATCA	8003.2	G	GGGACGAATACGCATATTGCATCAC	8250.4
HEV	AAAGGAAACACGGACA	4941.3	G	AAAGGAAACACGGACAC	5188.4
H7	ATGGACAAACTGTACGA	5227.4	A	ATGGACAAACTGTACGAA	5498.6
H3N2 seasonal	AATCTTCCTGTATGCTCA	5424.5	A	AATCTTCCTGTATGCTCAA	5695.8
HBoV	CTCTATATTGAAGGATCTGCAT	6724.4	C	CTCTATATTGAAGGATCTGCATG	7011.6
GAPDH	ATACGACCAAATCTAAGAGAC	6416.3	G	ATACGACCAAATCTAAGAGACG	6769.6

### Clinical application of respiratory pathogen detection (MRT-PCR-MassARRAY)

The collected nasopharyngeal swab samples were tested with MRT-PCR-MassARRAY, as described above. All target respiratory viruses were screened using consensus PCR/reverse transcription (RT)-PCR assays, according to previous reports [16–25]. PCR was performed using the FastStart high-fidelity PCR system (Roche Molecular Systems, Inc., Pleasanton, CA, USA). RT-PCR was performed using the One-Step RT-PCR kit (Invitrogen). Amplified DNA was purified using a QIAquick gel extraction kit, according to the manufacturer’s protocol (Qiagen, Valencia, CA, USA) and sequenced on an ABI3730 automated sequencer (Applied Biosciences, Foster City, CA, USA). Differences between the detection rates of the two methods were tested using the McNemar’s test. A *P* value of < 0.01 was considered statistically significant.

## Results

### Sensitivity and specificity of MRT-PCR-MassARRAY for respiratory pathogen

#### Detection

**Fig. 1:**
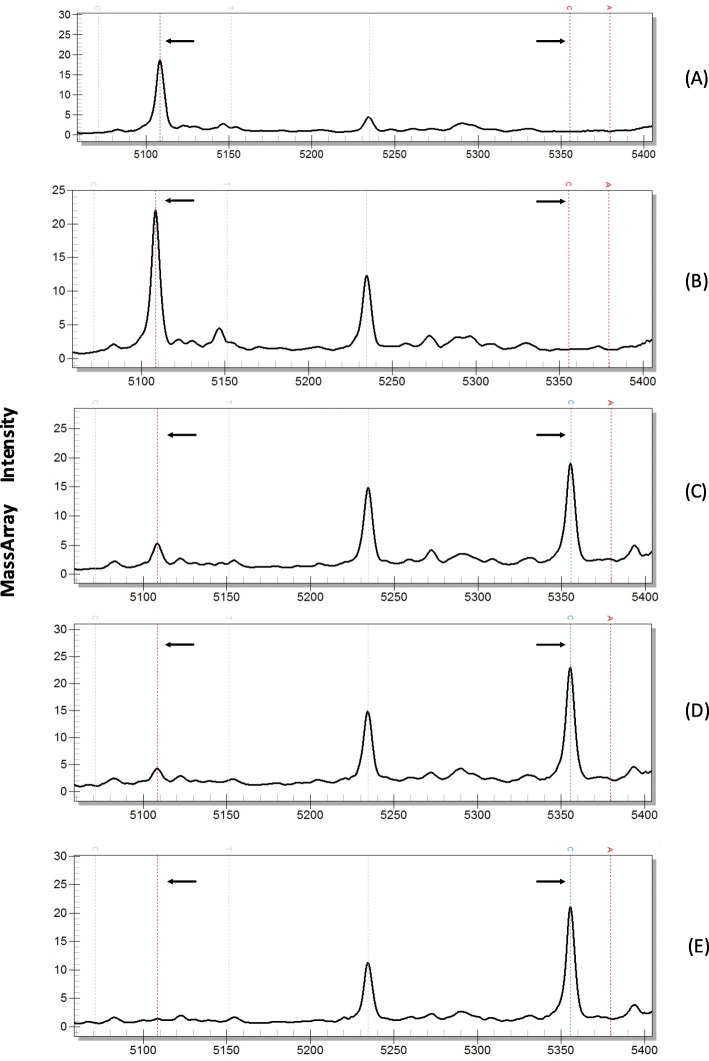
Mass spectrum results showing the sensitivity of the detection of *Mycoplasma pneumoniae*. **A** negative control, **B**–**E** *M pneumoniae* strain diluted to 10, 100, 1000, and 10,000 copies/μl, respectively. The arrow on the left side of the spectrum results indicates the position of the extension primer peak, the arrow on the right side indicates the position of the extension product, and the peak at approximate 5235 Da is the non-extended primer of HCoV. When concentrations of the negative control and pathogen were 10 copies/μl, there was only an extension primer peak and no extension product peak. At plasmid concentrations of 100, 1000, and 10,000 copies/μl, extension product peaks appeared, and the extension primer peaks became smaller or absent due to consumption. Thus, the detection sensitivity for *M. pneumoniae* was 100 copies/μl

For the negative control and pathogens below the detection limit, only the extension primer peak was detected but no extension product peak, while at dilutions above the detection limit, the extension primer is consumed, and only the specific extension product peak can be seen (Fig. 1). The analysis of the detected peaks of 27 pathogens showed only a single extension product for each pathogen, indicating that the specificity of the MRT-PCR-MassARRAY detection system was good. And the detection limit of all pathogens was between 10 copies/μl to 100 copies/μl (Table 3).

**Table 3:** Detection limit of the MRT-PCR-MassARRAY

Pathogens	LOD (copies/ul)	Pathogens	LOD (copies/ul)
*M.catarrhalis*	100	H1N1	100
HRV	100	*S.agalactiae*	10
MP	100	PIV3	100
HCoV	100	PIV2	100
Flu-A	100	ADV	100
CP	10	RSV	1000
Flu-B	1000	H-3	100
HMPV	100	*L. pneumophila*	100
*B.pertussis*	100	PIV1	10
*H. influenzae*	100	HEV	10
H-5	10	H-7	10
*S.pyogenes*	100	H3N2 seasonal	100
*S.pneumoniae*	100	HBoV	10
PIV4	10		

### Clinical performance of the MRT-PCR-MassARRAY detection system

Of the 207 clinical samples that were detected and analyzed using the MRT-PCR-MassARRAY detection system, 57 (27.54%) cases were positive, of which 33 cases were infected with a single pathogen, 19 cases with two pathogens, and five cases with three to four pathogens. *S. pneumoniae* was detected in 20 cases (9.66%), *M. catarrhalis* in 12 (5.80%), *M. pneumoniae* in 11 (5.31%), and *H. influenzae* in 1 (5.31%), PIV in ten (4.83%; including types 1–4 types), HMPV in seven (3.38%), and influenza virus type A in 6 (2.90%). The details of other detected pathogens are presented in Table 4.

**Table 4:** Detection of Each Pathogen

Pathogen	Single infection (*n* = 33)	Dual infections (*n* = 19)	Multiple infections (*n* = 5)	Total
ADV	0	1 (0.48%)	0	1 (0.48%)
BP	3 (1.45%)	3 (1.45%)	1 (0.48%)	7 (3.38%)
FluA	2 (0.97%)	3 (1.45%)	1 (0.48%)	6 (2.90%)
Hin	4 (1.93%)	5 (2.41%)	2 (0.97%)	11 (5.31%)
HCOV	0	1 (0.48%)	0	1 (0.48%)
HMPV	3 (1.45%)	1 (0.48%)	3 (1.45%)	7 (3.38%)
MC	3 (1.45%)	8 (3.86%)	1 (0.48%)	12 (5.80%)
MP	3 (1.45%)	4 (1.93%)	4 (1.93%)	11 (5.31%)
PIV-1	1 (0.48%)	0	1 (0.48%)	2 (0.97%)
PIV-2	0	1 (0.48%)	2 (0.97%)	3 (1.45%)
PIV-3	1 (0.48%)	1 (0.48%)	0	2 (0.97%)
PIV-4	2 (0.97%)	0	1 (0.48%)	3 (1.45%)
RSV	1 (0.48%)	1 (0.48%)	0	2 (0.97%)
SP	9 (4.35%)	9 (4.35%)	2 (0.97%)	20 (9.66%)
GAS	1 (0.48%)	0	0	1 (0.48%)

### Comparison of MRT-PCR MassARRAY and the consensus PCR/RT-PCR method

There were only two sample discrepancies in the test results between the two assays, both of which were positive by MassARRAY but negative by consensus PCR/RT-PCR. The positive, negative, and total correlation rates were 100, 98.68, and 99.03%, respectively. There was a high degree of agreement between the test results of the two methods (*P* < 0.01 by McNemar’s test).

## Discussion

The global incidence of respiratory tract infections is high, leading to millions of deaths annually. In one hospital, 36.7% of deaths in patients < 12 years old were due to pneumonia caused by respiratory tract infections [24]. The wide variety of pathogens and similar clinical symptoms of respiratory tract infections pose great difficulties to clinicians for diagnosis and treatment. In this study, we had developed a new method using multiplex RT-PCR combined with mass spectrometry to detect common pathogens infected in respiratory tract, and also we had evaluated its analytical and clinical performace.

Of the 27 pathogens in our study, only influenza B virus and respiratory syncytial virus had a detection sensitivity of 1000 copies/μl, and the remainder was detected at a sensitivity of 100 copies/μl. Thus, the RT-PCR MassARRAY method established in this study had high detection sensitivity. In the analysis of 207 clinical samples for the detection of respiratory tract pathogens, the positive detection rate was 27.54% (57/207). *S. pneumoniae*, *H. influenzae*, *M. pneumoniae*, and *M. catarrhalis* are the main pathogens of community-acquired pneumonia and acute bacterial infections [25], and the detection rates of these four pathogens were found to be higher than those of other bacteria, such as *Bordetella pertussis* and *Legionella pneumoniae*, in this study. It was also found that among the 20 cases of *S. pneumoniae* infection, there were only 9 (45.0%) cases of single pathogen infection, and among 11 cases of dual and multiple infections, there were five cases of *M. catarrhalis* co-infection and four of *H. influenzae* co-infection, which indicated that co-infections between bacteria were relatively common. Furthermore, the antibiotic resistance of these three bacteria is different [26]. Comprehensive and detailed pathogen diagnosis is of great significance in guiding the rational use of antibiotics to avoid the inappropriate prescription of antibiotics. There were 21/207 (10.1%) samples that tested positive for viruses (note: there were two samples co-infected by three viruses). In China, viral infections are highly prevalent in autumn and winter and relatively low in spring and summer [27]. The samples in this study were collected from clinical patients from April to July (during spring and summer), which may be one of the reasons for the low positive rate of viral respiratory tract infections. PIV was detected in ten cases, including two cases of PIV1, three of PIV2, two of PIV3, and three of PIV4. There was no significant difference in the number of subtypes, which was different from the positive detection rates of PIV types 1, 2, 3, and 4 reported by Wang et al. (2019) in Shanghai, of 2.74, 0.62, 8.59, and 3.40%, respectively [28]. This may be because that Wang et al.’s study population was mainly children, and PIV3 is one of the leading causes of lower respiratory tract infections in infants as well as immunocompromised people [29]. In this study, seven cases (3.38%) of HMPV were detected, which was close to the positive detection rate of HMPV of 3.53% reported in 2018 by Zhong et al. (2019) [30]. We also detected six (2.90%) cases of influenza A virus, all of which were H1N1. Additionally, respiratory syncytial virus was detected in two cases and adenovirus and human coronavirus in one case each. Due to the limited number of samples and sample types, some subtypes of human bocavirus; human enterovirus; influenza B; and influenzae A H3, H3N2 seasonal, H5, and H7 were not detected in this study.

The MRT-MassARRAY respiratory pathogen detection system established in this study detected 27 respiratory pathogens and showed sensitivity and specificity similar to the results obtained using consensus PCR/RT-PCR. The advantage over real-time PCR is mainly reflected in the multiplicity of nucleic acid mass spectrometry amplification. In this study, a 27-plex one tube amplification mode was used, and the test cost was as low as 15 dollars per sample. MassARRAY detection uses 96/384 chips, with a high throughput of up to 384 samples. Based on this feature, our detection system is highly suited to early clinical pathogen screening and regional respiratory pathogen epidemiological investigations. A potential concern of this detection system is that a SNP could occur at the extension position which will lead to a nonspecific peak arising in the mass window, with its molecular weight not being consistent with the molecular weight in Table 2. Actually, this is a very rare situation that we had not encountered in this study, in case it happens Sanger sequencing of the PCR product would have to be done to determine the exact pathogen.

## Conclusion

In summary, the analytical and clinical performance of the developed system based on MassARRAY technology for detecting 27 common respiratory pathogens is reliable and showing superior sensitivity, specificity, high throughput and user-friendly handling compared with other methods. It is a useful and cost-effective method, which have a good potential to be implemented for screening of respiratory pathogens and is of great significance in the clinical diagnosis of respiratory disease. Finally, large-scale population verification for the performance of MRT-PCR-MassARRAY will facilitate its clinical application.

## Data Availability

The data that support the findings of this study are available from the corresponding author, upon request.
